# Supplementary material for: Women’s self-help group participation and discussion of reproductive coercion: Associations with past experiences of violence among family planning clients in Kenya
Source: PLOS Glob Public Health. 2025 Sep 10;5(9):e0004087. doi: 10.1371/journal.pgph.0004087 (PMC12422437; doi:10.1371/journal.pgph.0004087)
Supplement: Attachment — Submitted filename: Response to Reviewers.docx [file pgph.0004087.s002.docx]

June 1, 2025

UC San Diego Health

Department of Medicine

Division of Global Public Health and Infectious Disease

9500 Gilman Dr,

La Jolla, CA 92093

Jasmine Uysal, MPH,

Pre-doctoral fellow

Center on Gender Equity and Health

Department of Medicine

UC San Diego

Drs. Catherine Kyobutungi and Madhukar Pai

Editorial office, PLOS Global Public Health

Drs. Catherine Kyobutungi and Madhukar Pai,

Thank you for the opportunity to revise and resubmit manuscript # PGPH-D-24-02696, now entitled, “Women’s Self-Help Group Participation and Discussion of Reproductive Coercion: Associations with Past Experiences of Violence Among Family Planning Clients in Kenya”, for consideration as a research article in PLOS Global Public Health.

Below I have responded to the minor revisions requested.

Reviewer #1: The title seems clunky, repetitive and not especially helpful for understanding what the study is about. SUGGESTED TITLE: Experiences and discussions of reproductive coercion and intimate partner violence among participants in women-only self-help groups in Kenya: Findings from the ARCHES Baseline Survey

*Response: The authors thank the reviewer for this helpful reflection. The authors agree with the reviewer that the title could be improved. However, the suggestion offered by the reviewer seems to suggest a few attributes of the paper may be misleading, for example the sample is not among women in self-help groups and we did not measure discussion of IPV, only RC. Instead, the authors propose: “Women’s Self-Help Group Participation and Discussion of Reproductive Coercion: Associations with Past Experiences of Violence Among Family Planning Clients in Kenya.” We believe this version better focuses on women’s self-help group participation, discussion of RC only and that the sample is among FP clients, while reducing length and improving overall clarity.*

Reviewer #1: In the introduction, can you describe the nature of the association you examined between experiences of RC and IPV and self-help group participation? What was the temporality of relationship from the perspective of study authors? People who experience these things seek out support from self-help groups? Or participation in self-help groups leads to these experiences?

*Response: We appreciate the reviewer’s question about the nature and temporality of the association examined. As this is a cross-sectional analysis, we are not able to determine causal direction. Our objective was not to test whether participation in self-help groups (SHGs) reduces risk of reproductive coercion (RC) or intimate partner violence (IPV), nor whether experiences of RC/IPV drive SHG participation. Rather, our aim was to assess whether SHGs may be a viable context for offering support on these issues—particularly whether women experiencing RC and IPV are already present in these groups and whether they are using them to discuss such experiences.*

*Given existing concerns that women experiencing RC/IPV may face mobility or autonomy constraints that could prevent their participation in SHGs, we were interested in whether participation was systematically lower among those with prior experiences of violence. Conversely, we also explored whether those who had experienced RC/IPV were more likely to discuss these topics within SHGs, suggesting a possible organic use of these groups as spaces for support. While we cannot determine temporality, the findings offer insight into whether SHGs may already serve as entry points for supportive programming, or whether barriers exist that would need to be addressed for future intervention.*

*The authors have made revisions in the last paragraph of the introduction to clarify this.*

Reviewer #2: Reviewer #2: It is a much needed research, cause I am not an expert in your methodology. However, this paper needs to be reviewed by an expert in the methodology needed.

*Response: The authors thank the reviewer for recognizing the importance of this work.*

Editor: The manuscript offers helpful insights into relationships between female participation in self-help groups and discussions of reproductive coercion and intimate-partner violence. The implications for intervention development are also explained well. However, I agree with Reviewer 1's suggestions for edits that would strengthen the clarity of the manuscript.

*Response: The authors thank the editor for the opportunity to address these comments and further clarify the manuscript. We have made suggestions as indicated by reviewer #1.*

Thank you for your consideration. We look forward to your feedback.

Sincerely,

Jasmine Uysal, Corresponding Author
