# Supplementary material for: Women’s self-help group participation and discussion of reproductive coercion: Associations with past experiences of violence among family planning clients in Kenya
Source: PLOS Glob Public Health. 2025 Sep 10;5(9):e0004087. doi: 10.1371/journal.pgph.0004087 (PMC12422437; doi:10.1371/journal.pgph.0004087)
Supplement: Attachment — Submitted filename: Response_to_Reviewers_auresp_2.docx [file pgph.0004087.s003.docx]

August 5, 2025

UC San Diego Health

Department of Medicine

Division of Global Public Health and Infectious Disease

9500 Gilman Dr,

La Jolla, CA 92093

Jasmine Uysal, MPH,

Pre-doctoral fellow

Center on Gender Equity and Health

Department of Medicine

UC San Diego

Drs. Catherine Kyobutungi and Madhukar Pai

Editorial office, PLOS Global Public Health

Drs. Catherine Kyobutungi and Madhukar Pai,

Thank you for the opportunity to revise and resubmit manuscript # PGPH-D-24-02696, now entitled, “Women’s Self-Help Group Participation and Discussion of Reproductive Coercion: Associations with Past Experiences of Violence Among Family Planning Clients in Kenya”, for consideration as a research article in PLOS Global Public Health.

Below I have responded to the minor revisions requested.

Editor comments: The reviewers and I found the manuscript to be improved, with just a few remaining questions to be addressed in the methods and results. Once these are addressed, the manuscript should be set.

*Response: Thank you for the thoughtful feedback. We have addressed the remaining questions below and made the corresponding clarifications in the manuscript.*

Reviewer #1: Looks good - thanks.

*Response: Thank you for your review.*

Reviewer #3: The paper is both descriptive and inferential. The investigators used descriptive statistics, bivariate hypotheses testing, and adjusted mixed effect logistic regression models to examine the relationship between self-help group participation and lifetime experience of reproductive coercion and intimate partner violence. For women who had participated in self-help groups within the past three months, they used these same methods to analyze group discussions about reproductive coercion in relation to lifetime experiences of abuse. The presentation is fairly well written from a statistical perspective. However, there are a few issues to be clarified.

*Response: Thank you for this thoughtful summary and feedback. We have addressed your specific questions below and made minor revisions to the manuscript where indicated.*

Reviewer #3: Why 659 subjects? There obviously was no formal calculation justifying this. The Investigators do admit possible limited power as a limitation. That may or may not be as the confidence intervals on Tables 3 and 4 look reasonable.

*Response: This study is a secondary analysis of baseline data from a pilot, non-randomized, cluster-controlled trial. Power calculations were conducted as part of the parent study design and are available in the cited parent study protocol. We have clarified this in the first paragraph of the Methods section (page 6).*

Reviewer #3: Also see the first column of Table 1. How representative is this sample to the same age group of women in Kenya or Nairobi?

*Response: This is a convenience sample of women attending private clinics during the intervention period and is not intended to be representative of all women of reproductive age in Kenya or Nairobi. We have added this clarification in the Methods section (page 6) and the limitations (page 15).*

Reviewer #3: Why private clinics? See the methods section. There should be some discussion of this unless this reviewer missed it.

*Response: The private clinics included in this study were operated by Family Health Options Kenya (FHOK), the local implementing partner for the parent evaluation study. Their participation was determined by the scope and design of the parent intervention. We have clarified this in the Methods section (page 6).*

Reviewer #3: The statistics are done appropriately in both the univariate and multivariate approaches. The limitations are clearly stated, a major one being that the this is a cross-sectional study based on retrospective self-report. Was there any follow up validation of any sample of the reports?

*Response: No, there was no follow-up validation of participant responses. All data were self-reported. We have clarified this in the limitations paragraph of the Discussion (page 15).*

Thank you for your consideration. We look forward to your feedback.

Sincerely,

Jasmine Uysal, Corresponding Author
